# Joint effects of paraoxonase 1 rs662 polymorphism and vitamins C/E intake on coronary artery disease severity (Gensini and SYNTAX scores) and lipid profile in patients undergoing coronary angiography

**DOI:** 10.3389/fnut.2022.1097411

**Published:** 2023-02-02

**Authors:** Mina Darand, Amin Salehi-Abargouei, Mohammad Yahya Vahidi Mehrjardi, Awat Feizi, Seyed Mustafa Seyedhossaini, Gholamreza Askari

**Affiliations:** ^1^Department of Community Nutrition, School of Nutrition and Food Sciences, Isfahan University of Medical Sciences, Isfahan, Iran; ^2^Nutrition and Food Security Research Center, Isfahan University of Medical Sciences, Isfahan, Iran; ^3^Nutrition and Food Security Research Center, Shahid Sadoughi University of Medical Sciences, Yazd, Iran; ^4^Department of Nutrition, School of Public Health, Shahid Sadoughi University of Medical Sciences, Yazd, Iran; ^5^Yazd Cardiovascular Research Center, Non-Communicable Disease Research Institute, Shahid Sadoughi University of Medical Sciences, Yazd, Iran; ^6^Research Center for Food Hygiene and Safety, School of Public Health, Shahid Sadoughi University of Medical Sciences, Yazd, Iran; ^7^Department of Biostatistics and Epidemiology, School of Health, Isfahan University of Medical Sciences, Isfahan, Iran

**Keywords:** PON1, Q192R polymorphism, rs662, lipid profile, coronary artery disease, vitamin C, vitamin E

## Abstract

**Introduction:**

Considering the emergence of the concept of personalized nutrition in recent years and its importance in the treatment of diseases, the purpose of this study was to investigate the interaction of paraoxonase (PON)1 rs662 polymorphism and vitamin C/E intake on coronary artery disease (CAD) severity and lipid profile in patients undergoing diagnostic angiography.

**Methods:**

This cross-sectional study was carried out on 428 patients undergoing angiography. The PON-1 genotypes were detected by the polymerase chain reaction-restriction fragment length polymorphism technique. Dietary intake was obtained using a valid questionnaire.

**Results:**

After adjustment for potential confounders, R allele carriers (RR + RQ) have lower HDL-C levels than non-carriers (QQ) (*P* ≤ 0.05). On the other hand, higher consumption of vitamin C was associated with a reduced risk of high total cholesterol (OR: 0.42, 95% CI 0.23–0.75, *P* = 0.003) and low-density lipoprotein cholesterol (OR: 0.49, 95% CI 0.25–0.96, *P* = 0.038) and an increased risk of low high-density lipoprotein cholesterol (OR: 1.88, 95% CI 1.03–3.42, *P* = 0.037). Furthermore, a significant interaction was observed between vitamin C intake and genotypes of rs66 polymorphism on LDL-C (*P* = 0.050). In detail, the R-allele carriers with lower vitamin C intake had higher LDL-C levels than QQ genotype carriers. No significant interaction was found between vitamin E intake and rs662 polymorphism genotypes on the Gensini and SYNTAX scores and lipid profile (*P* > 0.05).

**Conclusion:**

The novel finding of the present study was the existence of a significant interaction between rs662 polymorphism and vitamin C intake on LDL-C. More specifically, R allele carriers with lower vitamin C intake were susceptible to higher LDL-C.

## Introduction

Coronary artery disease (CAD) is the third leading cause of disability and death worldwide, killing about 17.8 million people annually (1). The disease puts a heavy economic burden on healthcare services (2) and causes irreparable physical and mental damage to the individual (3). CAD is a complex multifactorial disease that results from the interaction of genetic background and environmental factors (4). Atherosclerosis is the main cause of CAD, chiefly caused by high total cholesterol (TC) and low-density lipoprotein cholesterol (LDL-C) levels and low high-density lipoprotein cholesterol (HDL-C) levels (5). Along with medical therapy, nutritional interventions are considered one of the main treatments for controlling risk factors (6). For example, several studies have shown that a higher intake of antioxidant vitamins E and C has a protective effect against lipid disorders and as a result, CAD (7–9). On the other hand, the role of genetic factors in CAD has been well documented (10, 11). One of the genes whose association with CAD and lipid profile has been extensively investigated is the high-polymorphic paraoxonase 1 (PON1), which is located on the long arm of chromosome 7 (q21.3–22.1). Several meta-analyses have shown that the PON1 rs662 polymorphism (Q192R) is highly associated with CAD and its risk factors (12–14). Although the role of nutrition and genetic background in the development and treatment of CAD is well known, the results of studies are contradictory, which may be due to not considering the interaction of genetic and environmental factors (e.g., nutrition). In the new concept of gene-diet interaction, the different metabolic responses of people to active dietary molecules are justified and can be attributed to genetic differences (15). So far, few studies have been conducted on the interaction of PON1 rs662 polymorphism with dietary components (16). In a study on ischemic stroke, an interaction between vegetable consumption and rs662 was observed. Carriers of the A allele benefited more from consuming vegetables and were more effectively protected from stroke than carriers of the G allele (16). In another study, an interaction between folate intake and PON1 rs662 polymorphism was seen in the white population. In this study, the risk of incident ischemic stroke increased with increasing folate intake in the R allele carriers (17).

Although the independent association of PON1 rs662 polymorphism as well as vitamin E and C intake with the risk of CAD and lipid profile, has been investigated in previous studies, there are still disagreements that could be caused by gene-dietary components interaction. In other words, although vitamins C and E have an antioxidant nature, the response of all individuals to them is not the same, and their higher consumption may increase the risk of disease in some genetic variants. Due to the importance of individual nutrition in recent years and the lack of investigation of the simultaneous effect of the rs662 polymorphism and vitamin C/E intake, the present study was conducted to investigate the interaction of PON1 rs662 polymorphism with vitamin C/E intake on CAD severity and lipid profile in patients undergoing diagnostic angiography.

## Materials and methods

### Participants

The present cross-sectional study was approved by the ethics committee of Isfahan University of Medical Sciences (Ethical approval code: IR.MUI.RESEARCH.REC.1400.200), Iran and was part of a larger research that its protocol was approved by the ethics committee of Shahid Sadoughi University of Medical Sciences, Yazd, Iran. Patients aged 25–75 years with increasing chest pain (angina) or acute-onset angina, who were identified as candidates for angiography after the initial examination (exercise stress testing or electrocardiogram (ECG) or computed tomography (CT) scan) by a cardiologist, were included in the study. Eventually, among the patients admitted under diagnostic coronary angiography in Afshar Hospital in Yazd, based on inclusion and exclusion criteria, 463 patients were recruited. Patients with the following criteria were not included in the study: (1) history of cancer, heart failure, heart attack, percutaneous coronary intervention (PCI), coronary artery bypass grafting (CABG), chronic kidney disease stage 3 and above, specific liver disease or receiving medication for liver disorders, immunodeficiency, AIDS; (2) people with severe obesity (body mass index (BMI) above 40); (3) pregnant and lactating women; (4) people who for any reason have limited food intake by mouth; (5) have a special diet. Non-response to many of food frequency questionnaire items and not detecting the type of genotype led to the patient's exclusion from the study. Finally, data from 428 persons were analyzed (Supplementary material: participant flowchart). Written informed consent was taken from eligible persons. The study was carried out per the Declaration of Helsinki.

### Assessment of the CAD severity

The severity of CAD was determined by Gensini and SYNTAX scores. For this purpose, the coronary angiogram was interpreted by an experienced cardiologist blinded for demographic and clinical data except for sex and age. Gensini and SYNTAX scores were computed randomly for several patients by the second cardiologist. The Gensini score calculation starts by allocating a severity score to each identified coronary stenosis as follows: 1 point for ≤25% narrowing, 2 points for 26–50% narrowing, 4 points for 51–75% narrowing, 8 points for 76–90% narrowing, 16 points for 91–99% narrowing, and 32 points for total occlusion (100%). After that, each lesion score is multiplied by a factor that takes into account the importance of the coronary arteries and the lesion's position in the coronary circulation (5 for the left main coronary artery, 2.5 for the proximal segment of the left anterior descending coronary artery, 2.5 for the proximal segment of the circumflex artery, 1.5 for the mid-segment of the left anterior descending coronary artery, 1.0 for the right coronary artery, the distal segment of the left anterior descending coronary artery, the posterolateral artery, and the obtuse marginal artery, and 0.5 for other segments). Finally, the Gensini score was acquired by summating of the coronary segment scores. A higher Gensini score demonstrates a more intensive disease (18–20). The patients were categorized into two groups based on Gensini score: low Gensini score (< 20) and intermediate-high Gensini score (≥ 20) (21).

The SYNTAX score was computed through the internet-based SYNTAX calculator version 2.0 (www.syntaxscore.com). SYNTAX score algorithm containing consecutive and interactional self-guided questions focusing on functional and anatomical parameters of the lesions with ≥50 % stenosis in arteries with a diameter of ≥1.5 mm. The final SYNTAX score was acquired by summation of all lesion scores. The participants were classified into two groups based on SYNTAX score: low SYNTAX score (<23) and intermediate-high SYNTAX score (≥23). A higher SYNTAX score represents a more intensive disease (22, 23).

### Dietary intakes assessments

The usual dietary intakes of patients during the past year were evaluated using the valid and reliable 182-item semi-quantitative food frequency questionnaire (FFQ) and face-to-face interviews with trained nutritionists. The FFQ used in this study was a modified version of a 178-item FFQ, which was previously validated to assess the dietary intake of Yazidis adults (24). Patients were asked to report the frequency (number of times per month, week, or day the food was consumed) and the amount of each food item that was consumed every time in the past year (portion size based on the standard serving sizes commonly consumed by Iranians). The reported values of each food item were converted to g/day using household measures of consumed foods. Then, nutrient intake was calculated using Nutritionist IV software (25).

### Anthropometric and blood pressure measurements

In this study, a nutritionist measured weight using a portable digital scale and the body analyzer (Omron Inc. Osaka, Japan), with an accuracy of 0.1 kg, with minimal coverage and without shoes.. Height was measured with an accuracy of 0.1 cm, using a wall-fixed measuring tape in a standing position with shoulders in normal alignment and no shoes. BMI was calculated as body weight (kg) divided by height squared (m^2^). Waist circumference was assessed by a flexible inelastic tape measure (i.e., the tape measure should not stretch when taking the measurement) in the standing position to the nearest 1 cm. The narrowest area between the iliac crest and the last rib was measured (26). The percentage of muscle mass, total body and visceral fat were also assessed using a body composition analyzer (Omron, Japan model: BF511) (27). Blood pressure was also measured using a bedside automated monitor, and the patient's average blood pressure during the day was recorded by nurses working at the cardiovascular care unit (CCU) before patients underwent angiography.

### Biochemical assessment

To determine the levels of triglyceride (TG), TC, HDL-C and LDL, after 10–12 h of overnight fasting, a 4 ml blood sample was drawn from the patients. 2 ml of blood samples were centrifuged at 2,500 rpm for 3 mins to separate the serum from the blood cells. Buffy coats and remaining whole blood samples were stored at −80°C for DNA extraction and other biochemical tests. TG, TC, LDL-C and HDL-C concentration are measured using Pars Azmon company kits in the Yazd School of Health laboratory. Then, lipid profile were categorized into normal TG (<150 mg/dl) or high TG (≥150 mg/dl), normal TC (<200 mg/dl) or high TC (≥200 mg/dl), normal LDL-C (<130 mg/dl) or high LDL-C (≥130 mg/dl), normal HDL-C (≥40 mg/dl for men and ≥50 mg/dl for women) or low HDL (<40 mg/dl for men and <50 mg/dl for women), per the Adult Treatment Panel III (28) and the 2013 American College of Cardiology/American Heart Association guidelines (29).

### DNA extraction and genotyping

DNA samples were isolated from the white blood cell genome of the complete blood sample of the participants using the SimBiolab Blood Kit, according to the manufacturer's protocol. The rs662 polymorphism (major allele: Q, minor allele: R), a fragment of 520 base pairs (bp) in exon 6 of the PON1 gene, was genotyped by the polymerase chain reaction-restriction fragment length polymorphism (PCR-RFLP) method. The PCR mixture was provided in a total volume of 20 μl containing 2 μl of genomic DNA, 10 μl of Master Mix (Amplicon, Denmark), 6 μl of water and 1 μl (10 pmol) of each oligonucleotide primer. Forward and reverse primer consists of AAACCCAAATACATCTCCCAGAAT and GCTCCATCCCACATCTTGATTTTA, respectively. PCR is performed by repeating three steps. First, DNA templates were denatured at 95°C for 5 min; amplification consisted of 45 cycles at 95°C for 15 s, annealing at 60°C for 30 s, extension at 72°C for 30 s, with a final extension at 72°C for 5 min. Amplified DNA (10 ml) was digested with 5 U restriction enzyme HinfI (Fermentase, Germany) at 37°C, overnight. All products were visualized by electrophoresis in 2% agarose gel (SinaClon, Iran) at 90 V for 2.5 h.

### Assessment of other variables

General demographic data including age, gender, smoking status, multivitamin intake, vitamin C and E supplements intake, the medication used, and medical history were collected using valid and reliable questionnaires. Physical activity was assessed using International Physical Activity Questionnaire (IPAQ). Physical activity level was calculated based on metabolic equivalent task—minutes per week (30). Persian translation validation of IPAQ has previously been confirmed by Moghaddam et al. (31).

### Statistical analysis

Subjects were divided into two genotype groups: R-allele carriers (QR/RR) and non-carriers (QQ). Pearson's chi-square test was used for the Hardy-Weinberg Equilibrium (HEW). Vitamin C and E intake was categorized into two categories based on median intake (higher and lower than the median). Gensini score, SYNTAX score, TG, TC, LDL-C and HDL-C were also categorized into two categories. Continuous and categorical variables were expressed as mean ± standard deviation (SD) and frequencies (percentages), respectively. The normality distribution of continuous variables was checked by the Kolmogorov- Smirnov test. Chi-squared test and independent samples t-test were used for comparing baseline categorical and continuous variables between genotypes (QQ, QR + RR) and categories of vitamin intake, respectively. The Gensini score, SYNTAX score, HDL-c, LDL-c, TC and TG as both categorical and continuous response variables based on conducted statistical analysis were treated. The covariance (ANCOVA) test under the generalized linear model (GLM) framework was used to analyze the independent and interaction effects of PON1 rs662 genotypes and vitamin intake on Gensini and SYNTAX scores and lipid profile. The adjusted means were estimated after controlling for age, gender, total energy intake, physical activity, smoking status, alcohol consumption, multivitamin intake, vitamin C and E supplements intake, BMI and medication used (antihypertension drugs, antidiabetic drugs and antihyperlipidemic drugs). Logistic regression was used to examine the joint effect of vitamin C/E intake and rs662 genotypes (QQ and QR/RR) for predicting being at higher risk of CAD and lipid disorders. The association analyses were done in adjusted logistic regression models and adjustment was made for age, gender, total energy intake, physical activity, smoking status, alcohol consumption, BMI, multivitamin intake, vitamin C and E supplements intake and medication used (antihypertension drugs, antidiabetic drugs, and antihyperlipidemic drugs). The analyses were performed using SPSS software version 24 (IBM Corp., Armonk, NY, USA). *P* ≤ 0.05 were considered significant.

## Results

### Study population characteristics

Data from 428 patients (aged 25–75 years) were analyzed. The prevalence of PON1 rs662 polymorphism genotypes was QQ (47.4%), QR + RR (52.6%). Table 1 presents the characteristics and dietary intake of study participants according to PON1 genotypes and vitamin C and E intake. The mean intake of energy and fat was higher in R-allele carriers, while the average carbohydrate intake was lower than in the non-carriers. Age, BMI, gender, physical activity, medication used (antihypertension and antihyperlipidemic drugs), smoking status, alcohol consumption, mean energy intake, carbohydrate, fat, cholesterol and vitamin C supplement intake was different between the two categories of vitamin E intake. Vitamin E consumption was higher in younger people with higher physical activity. On the other hand, vitamin E intake was higher in men than in women. Also, the average energy and fat intake was higher in people with higher vitamin E intake. On the other hand, BMI, gender, smoking, alcohol consumption, and dietary intake were significantly different in the two categories of vitamin C intake. In people with a higher intake of vitamin C compared with individuals who received less than the median, the consumption of energy, carbohydrates, and folate was higher, while the cholesterol and saturated fatty acids intake were lower.

**Table 1:** Characteristics and dietary intake of study participants according to PON1 genotypes and two categories of vitamin E and C intake.

**Variables**	**Type of genotype**			**Vitamin E**			**Vitamin C**		
	**QQ**	**QR/RR**	*P* ^a^	**Low (**<**11.20)**	**High (**>**11.20)**	*P* ^a^	**Low (**<**194.45)**	**High (**>**194.45)**	*P* ^a^
**Age (years)**	57.20 ± 9.61^b^	56.33 ± 9.19	0.344	58.19 ± 8.98	55.31 ± 9.58	0.002	56.39 ± 9.64	56 ± 9.10	0.158
**BMI (kg/m** ^**2**^ **)**	27.38 ± 4.06	27.54 ± 4.55	0.699	27.76 ± 4.65	27.17 ± 3.96	0.167	27.88 ± 4.53	27.03 ± 4.07	0.048
**Physical activity (Met_min/week)**	3,833 ± 6,619	4,368 ± 7,884	0.452	3,357 ± 5,909	4,886 ± 8,442	0.032	3,597 ± 6,312	4,630 ± 8,154	0.145
**Gender, male**, ***n*** **(%)**	124 (46.1.1)	145 (53.9)	0.436	120 (56.1)	150 (70.1)	0.003	113 (52.8)	157 (73.4)	<0.0001
**Antihypertension drugs, yes**, ***n*** **(%)**	89 (47.1)	100 (52.9)	0.900	113 (52.8)	76 (35.5)	<0.0001	95 (44.4)	94 (43.9)	0.922
**Antidiabetic drugs, yes**, ***n*** **(%)**	63 (31)	75 (33.3)	0.611	74 (34.6)	64 (29.9)	0.301	64 (29.9)	74 (34.6)	0.301
**Antihyperlipidemic drug, yes (%)**	76 (37.4)	77 (34.2)	0.488	93 (43.5)	60 (28)	0.001	80 (37.4)	73 (34.1)	0.480
**Alcohol consumption**, ***n*** **(%)**			0.891			0.062			0.009
Never	190 (94.5)	209 (93.7)		205 (96.7)	194 (91.5)		206 (97.6)	193 (90.6)	
Current consumption	7 (3.5)	8 (3.6)		5 (2.4)	10 (4.7)		3 (0.9)	12 (5.6)	
Former consumption	4 (2)	6 (2.7)		2 (0.9)	8 (3.8)		2 (0.9)	8 (3.8)	
**Smoking status**, ***n*** **(%)**			0.347			<0.0001			<0.0001
Never smoker	139 (50)	139 (50)		164 (59)	114 (41)		159 (74.3)	119 (55.6)	
Current smoker	57 (42.9)	76 (57.1)		42 (31.6)	91 (68.4)		47 (22)	86 (40.2)	
Former smoker	7 (41.2)	10 (58.8)		8 (47.1)	9 (52.9)		8 (3.7)	9 (4.2)	
**Vitamin E supplements intake**, ***n*** **(%)**			0.456			0.517			0.778
Never	192 (94.6)	209 (92.9)		203 (94.9)	198 (92.5)		202 (94.4)	199 (93)	
1–3/month	6 (3)	5 (2.2)		4 (1.9)	7 (303)		4 (1.9)	7 (3.3)	
Minimal once a week	3 (1.5)	4 (1.8)		2 (0.9)	5 (2.3)		3 (1.4)	4 (1.9)	
Minimal once a day	2 (1)	7 (3.1)		5 (2.3)	4 (1.9)		5 (2.3)	4 (1.9)	
**Vitamin C supplements intake**, ***n*** **(%)**			0.674			0.019			0.103
Never	162 (79.8)	189 (84)		176 (82.2)	175 (81.8)		176 (82.2)	175 (81.8)	
1–3/month	14 (6.9)	14 (6.2)		20 (9.3)	8 (3.7)		19 (8.9)	9 (4.2)	
Minimal once a week	17 (8.4)	13 (5.8)		13 (6.1)	17 (7.9)		11 (5.1)	19 (8.9)	
Minimal once a day	10 (4.9)	9 (4)		5 (2.3)	14 (6.5)		8 (3.7)	11 (5.1)	
**Multivitamin intake**, ***n*** **(%)**			0.248			0.433			0.829
Never	187 (92.1)	208 (92.4)		200 (93.5)	195 (91.1)		200 (93.5)	195 (91.1)	
1–3/month	9 (4.4)	8 (3.6)		9 (4.2)	8 (3.7)		7 (3.3)	10 (4.7)	
Minimal once a week	4 (2)	1 (0.4)		1 (0.5)	4 (1.9)		2 (0.9)	3 (1.4)	
Minimal once a day	3 (1.5)	8 (3.6)		4 (1.9)	7 (3.3)		5 (2.3)	6 (2.8)	
**Dietary intake**									
Energy intake (kcal)	2,592 ± 1,101	2,897 ± 1,426	0.013	2,000 ± 742	3,504 ± 1,284	<0.0001	2,070 ± 840	3,435 ± 1,302	<0.0001
Protein	14.87 ± 0.03	15.14 ± 0.03	0.423	15.13 ± 0.03	14.90 ± 0.03	0.428	15.17 ± 0.03	14.83 ± 0.03	0.352
Carbohydrate	62.06 ± 0.08	60.14 ± 0.09	0.024	62.16 ± 0.08	59.94 ± 0.08	0.009	59.69 ± 0.09	62.42 ± 0.08	0.001
Fat	24.72 ± 0.06	26.11 ± 0.07	0.038	23.76 ± 0.06	27.14± 0.06	<0.0001	25.87 ± 0.07	25.03 ± 0.06	0.213
Cholesterol	177.21 ± 95.74	167.21 ± 67.39	0.209	186.73 ± 84.76	157.54 ± 76.92	<0.0001	179.96 ± 64.08	163.94 ± 96.36	0.044
Saturated fatty acid	8.28 ± 2.95	8.72 ± 3.05	0.131	8.53 ± 3.12	8.94 ± 2.90	0.897	8.83 ± 3.11	8.19 ± 2.87	0.028
Folate	169.34 ± 39.20	171.80 ± 60.08	0.620	167.56 ± 41.47	173.70 ± 59.31	0.212	156.89 ± 42.37	184.37 ± 55.52	<0.0001
Vitamin B12	1.48 ± 0.85	1.62 ± 1.14	0.151	1.63 ± 1.12	1.48 ± 0.89	0.121	1.62 ± 0.90	1.49 ± 1.11	0.193

BMI, body mass index.

Vitamin E and C intake is categorized into two categories, “high” and “low,” based on the median intake.

a Obtained from Chi-squared test and independent t-test for categorical and continuous variables respectively.

b Continuous and categorical data are presented as mean ± (SD) and frequency (percentage).

Protein, carbohydrate and fat are presented as a (% of total daily energy).

Other dietary intake are presented as the mg/1,000 Kcal.

P < 0.05 was considered as statistically significant.

### The effects of PON1 rs662 polymorphism, vitamin C/E intake and the interaction of the two variables on Gensini and SYNTAX scores and lipid profile markers

Table 2 shows the mean values of Gensini and SYNTAX scores and lipid profile across PON1 genotypes (QQ and QR/RR) based on higher and lower than median vitamin E and C intake. After adjustment for age, gender, total energy intake, physical activity, smoking status, alcohol consumption, multivitamin intake, vitamin C and E supplement intake, BMI, and medication used, R allele carriers (RR + RQ) have lower HDL-C levels than non-carriers (QQ) (*P* ≤ 0.05). On the other hand, a higher vitamin C intake was associated with lower LDL-C (*P* = 0.007) and HDL-C levels (*P* = 0.010). Vitamin E intake was not significantly associated with any studied variables (*P* ≥ 0.05).

**Table 2:** Mean values of CAD risk factors across PON1 genotypes (QQ and QR + RR) based on two categories of vitamin E and C intake.

**Variables**		**Vitamin E intake**							**Vitamin C intake**						
		**Low**		**High**		*P* ^a^ ^†^	*P* ^b^ ^†^	*P* ^c^ ^†^	**Low**		**High**		*P* ^a^ ^†^	*P* ^b^ ^†^	*P* ^c^ ^†^
	**QQ**	**QR/RR**	**QQ**	**QR/RR**				**QQ**	**QR/RR**	**QQ**	**QR/RR**				
Gensini score	Crude	35.54 ± 4.08	31.22 ± 4.22	31.73 ± 4.68	39.19 ± 3.92	0.712	0.625	0.167	35.49 ± 4.21	33.02 ± 4.15	32.04 ± 4.53	37.97 ± 4.05	0.683	0.860	0.323
	Model 1	32.97 ± 3.38	30.21 ± 4.48	33.13 ± 4.91	39.90 ± 4.27	0.637	0.335	0.263	36.99 ± 4.32	35.06 ± 4.26	28.72 ± 4.70	35.39 ± 4.45	0.576	0.431	0.313
SYNTAX score	Crude	10.81 ± 1.26	10.10 ± 1.32	9.50 ± 1.44	11.90 ± 1.21	0.519	0.854	0.238	10.35 ± 1.30	10.55 ± 1.28	10.12 ± 1.39	11.59 ± 1.25	0.524	0.757	0.630
	Model 1	10.54 ± 1.37	10.19 ± 1.40	10.30 ± 1.53	11.73 ± 1.33	0.686	0.697	0.503	11.01 ± 1.35	11.34 ± 1.33	9.84 ± 1.47	10.61 ± 1.39	0.679	0.544	0.871
TG	Crude	160.30 ± 8.45	154.12 ± 9.04	142.97 ± 9.46	155.99 ± 8.11	0.697	0.379	0.275	149.42 ± 8.51	159.14 ± 8.63	156.51 ± 9.42	151.32 ± 8.46	0.796	0.967	0.396
	Model 1	159.63 ± 8.97	162.16 ± 9.36	146.42 ± 9.72	144.92 ± 8.84	0.953	0.160	0.818	153.17 ± 8.69	164.79 ± 8.91	154.40 ± 9.63	140.96 ± 9.26	0.916	0.280	0.153
TC	Crude	210.36 ± 11.13	195.80 ± 11.90	183.81 ± 12.46	210.65 ± 10.68	0.596	0.613	0.074	198.10 ± 11.23	206.32 ± 11.40	199.16 ± 12.44	201.82 ± 11.18	0.639	0.882	0.810
	Model 1	203.08 ± 11.86	204.36 ± 12.37	186.11 ± 12.85	207.93 ± 11.68	0.313	0.639	0.375	198.27 ± 11.49	219.20 ± 11.77	193.10 ± 12.72	192.83 ± 12.24	0.368	0.254	0.360
LDL-C	Crude	100.13 ± 4.14	97.92 ± 4.42	95.36 ± 4.63	98.69 ± 3.97	0.896	0.642	0.520	100.87 ± 4.12	105.74 ± 4.18	94.51 ± 4.56	91.23 ± 4.10	0.851	0.015	0.339
	Model 1	97.96 ± 4.53	98.71 ± 4.73	96.85 ± 4.91	97.68 ± 4.46	0.875	0.845	0.993	101.30 ± 4.34	108.18 ± 4.44	93.71 ± 4.80	87.41 ± 4.62	0.947	**0.007**	0.133
HDL-C	Crude	52 ± 1.14	47.99 ± 1.22	47.77 ± 1.28	47.58 ± 1.09	0.078	0.051	0.109	51.33 ± 1.15	49.20 ± 1.16	48.65 ± 1.27	46.38 ± 1.14	0.065	0.021	0.952
	Model 1	51.34 ± 1.33	48.15 ± 1.28	48.34 ± 1.33	46.98 ± 1.21	**0.050**	0.158	0.444	51.22 ± 1.18	49.86 ± 1.21	48.60 ± 1.31	45.09 ± 1.26	**0.040**	**0.010**	0.370

TG, triglyceride; TC, cholesterol; HDL-c, high density lipoprotein cholesterol; LDL-c, low density lipoprotein cholesterol; FBS, fasting blood sugar; SBP, systolic blood pressure; DBP, diastolic blood pressure.

Vitamin E and C intake is categorized into two categories, “high” and “low”, based on the median intake.

Data are presented as mean ± standard error (SE).

† Obtained from Generalized linear models (GLM).

P^a^: Association of genotypes and CAD risk factors: Genotypes main effect.

P^b^: Association of vitamins and CAD risk factors: Vitamin main effect.

P^c^: Interaction: vitamins and genotype interaction on CAD risk factors.

Model 1 is adjusted for age, gender, total energy intake, physical activity, smoking status, alcohol consumption, BMI, multivitamin intake, vitamin C and E supplements intake and medication use (antihypertension, antidiabetic and anti hyperlipidemic drugs).

Bolded values are statistically or marginally significant.

### The effect of the PON1 genotypes, vitamin C and E intake and the interaction of the two on CAD severity and lipid disorders

**Figure 1:**
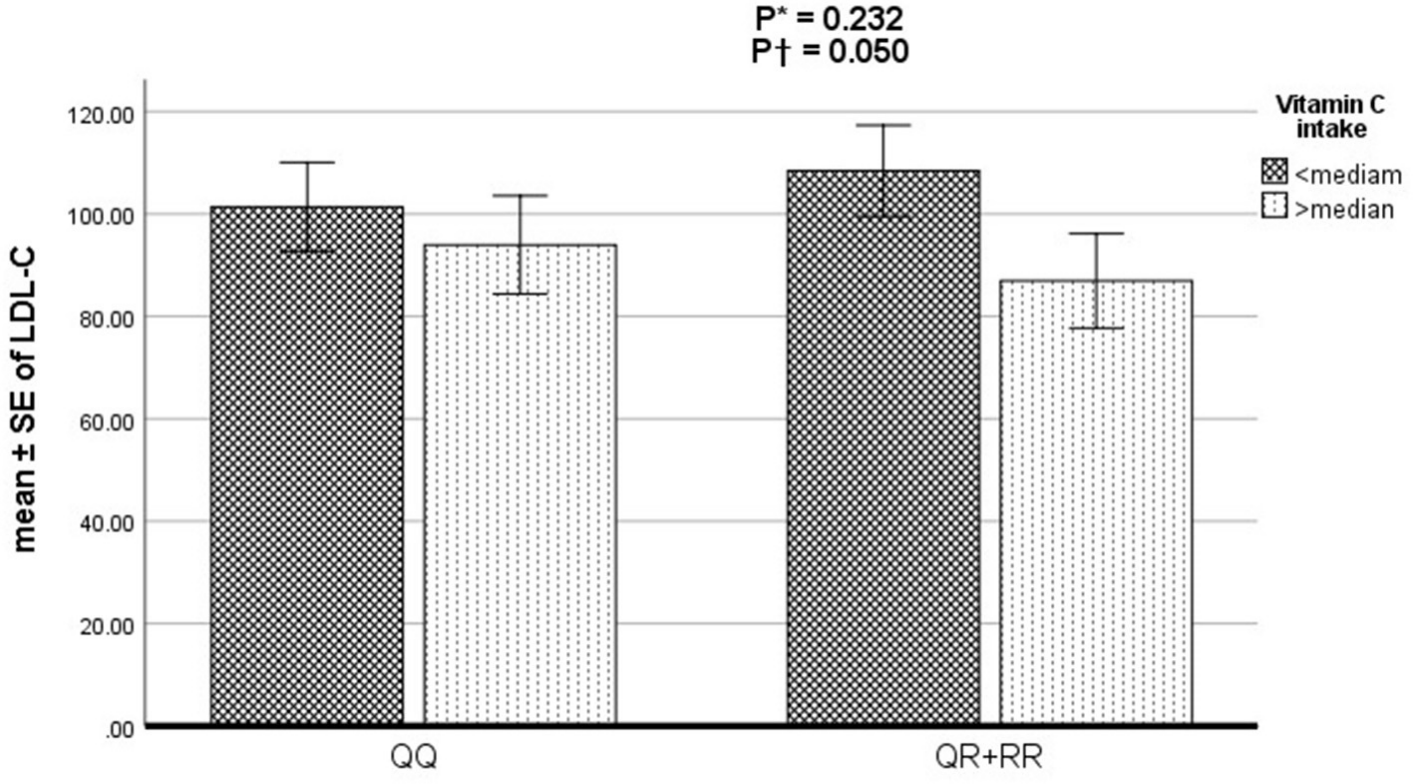
Interaction of PON1 rs662 polymorphism and vitamin C intake on serum LDL-C.

The odds of the association of vitamin C and E intake, PON1 rs662 genotypes (RR+RQ vs. QQ), and the interaction of the two variables with CAD severity and lipid disorders based on adjusted logistic regression are reported in Table 3. After adjustment for potential confounders, higher consumption of vitamin C was associated with a reduced risk of high TC (OR: 0.42, 95% CI 0.23–0.75, *P* = 0.003) and LDL-C (OR: 0.49, 95% CI 0.25–0.96, *P* = 0.038) and an increased risk of low HDL-C (OR: 1.88, 95% CI 1.03–3.42, *P* = 0.037). Furthermore, a significant interaction was observed between vitamin C intake and genotypes of rs66 polymorphism on LDL-C (*P* = 0.050) (Figure 1). In detail, the R-allele carriers with lower vitamin C intake had higher LDL-C levels than QQ genotype carriers. No significant interaction was found between vitamin E intake and rs662 polymorphism genotypes on the Gensini and SYNTAX scores and lipid profile (*P* > 0.05).

**Table 3:** The odds of CAD severity and its risk factors in terms of vitamin E and C intake, PON1 rs662 genotypes, and the interaction of the vitamin intake and genotypes.

**Variables**		**High Gensini**	***P* ^†^**	**High syntax**	***P* ^†^**	**High TG**	***P* ^†^**	**High TC**	***P* ^†^**	**High LDL-C**	***P* ^†^**	**Low HDL-C**	***P* ^†^**
Genotypes	QQ	1	1	1	1	1	1
	RQ + RR	1.11 (0.71–1.75)	0.623	1 (0.57–1.74)	0.996	0.90 (0.55–1.48)	0.697	0.82 (0.52–1.30)	0.413	1.17 (0.68–1.99)	0.558	1.18 (0.72–1.91)	0.502
Vitamin E intake	Low intake	1	1	1	1	1	1
	High intake	0.97 (0.57–1.77)	0.971	1.30 (0.65–2.56)	0.449	0.72 (0.38–1.33)	0.296	0.83 (0.47–1.49)	0.549	1.11 (0.57–2.15)	0.753	1.65 (0.89–3.07)	0.112
Interaction of vitamin E and genotypes	QQ*low	1	0.221	1	0.918	1	0.632	1	0.821	1	0.838	1	0.316
	QQ*high	0.74 (0.35–1.56)		1.26 (0.51–3.08)		0.81 (0.36–1.82)		0.89 (0.42–1.86)		1.04 (0.43–2.47)		2.12 (0.95–4.72)	
	RR/RQ*low	0.85 (0.45–1.59)		0.96 (0.43–2.12)		1.03 (0.51–2.06)		0.87 (0.46–1.66)		1.10 (0.52–2.34)		1.49 (0.75–2.98)	
	RR/RQ*high	1.12 (0.55–2.27)		1.29 (0.54–3.06)		0.66 (0.30–1.44)		0.70 (0.−1.44)		1.28 (0.56–2.94)		1.92 (0.88–4.22)	
Vitamin C intake	Low intake	1	1	1	1	1	1
	High intake	0.83 (0.48–1.43)	0.513	0.94 (0.49–1.80)	0.856	0.95 (0.52–1.74)	0.890	0.42 (0.23–0.75)	**0.003**	0.49 (0.25–0.96)	**0.038**	1.88 (1.03–3.42)	**0.037**
Interaction of vitamin C and genotypes	QQ*low	1	0.306	1	0.808		0.220	1	0.165	1	**0.050**	1	0.677
	QQ*high	0.66 (0.32–1.33)		1 (0.43–2.35)		1.31 (0.60–2.86)		0.57 (0.28–1.18)		1.85 (0.91–3.78)		1.70 (0.79–3.69)	
	RR/RQ*low	0.89 (0.48–1.66)		1.06 (0.49–2.30)		1.20 (0.61–2.36)		1.11 (0.59–2.06)		1.85 (0.91–3.78)		1.07 (0.55–2.10)	
	RR/RQ*high	0.95 (0.47–1.91)		0.93 (0.39–2.22)		0.84 (0.38–1.85)		0.32 (0.15–0.69)		0.52 (0.21–1.28)		2.27 (1.04–4.95)	

HDL-c, high density lipoprotein cholesterol; LDL-c, low density lipoprotein cholesterol; TC, total cholesterol; TG, triglyceride.

Vitamin E and C intake is categorized into two categories, “high” and “low,” based on the median intake.

† Obtained from logistic regression.

All values are odds ratio (95% CI for OR) and adjusted for age, gender, total energy intake, physical activity, smoking status, alcohol consumption, BMI, multivitamin intake, vitamin C and E supplements intake and medication use (antihypertension, antidiabetic and anti hyperlipidemic drugs).

Bolded values are statistically or marginally significant.

## Discussion

As far as we know, the present study is the first study that addressed the interaction of PON1 rs662 polymorphism and antioxidant vitamins E and C intake on CAD severity (Gensini and SYNTAX scores) and lipid profile. Surprisingly, the results of the present study showed that higher vitamin C intake was associated with the risk of low HDL-C. Although this finding was not consistent with the results of previous studies, a recent study showed that a higher intake of antioxidant vitamins C and E blunt the clinical and angiographic benefits of simvastatin and niacin therapy on HDL-C. High intakes of these vitamins appear to prevent increases in HDL-C in response to HDL-raising drugs by lowering lipoprotein (A-I) in CAD patients (32). Our findings indicated that the higher vitamin C intake significantly reduced the risk of high cholesterol and high LDL-C. Although some studies have reported the protective effect of a higher dietary vitamin C intake on serum cholesterol and LDL-C (33–35), the results are still contradictory, and no effect has been seen in other studies (36–38). Since CAD and lipid disorders are multi-caused diseases (39), the inconsistency in the published studies can be attributed to the interaction of several factors, including genetic background, gender, and dietary components, which may not have been considered (40). On the other hand, although most previous studies supported the protective effect of vitamin E against lipid disorder (41–43), the present study did not show a significant association between vitamin E intake and lipid profile. In addition, the present findings did not demonstrate a significant effect of rs662 polymorphism or vitamin intake on CAD severity (Gensini and SYNTAX scores). Research on the association between rs662 polymorphism and the CAD severity based on Gensini and SYNTAX score is limited. In line with our study, two studies reported that PON1 rs662 polymorphism is not associated with the severity and extent of atherosclerosis (44, 45). The novel finding of the present study is the existence of a significant interaction between vitamin C intake and genotypes of rs662 polymorphism on LDL-C. In detail, the R-allele carriers placed in the lower category of vitamin C intake had higher LDL-C levels compared to QQ genotype carriers. Although no study addressed the interaction of rs662 polymorphism genotypes with vitamin E and C, one study investigated the interaction of rs662 polymorphism with vegetable and fruit consumption. In this study, rs662_R allele carriers who consume higher vegetables have a significantly lower risk of ischemic stroke and may benefit more effectively from the joint effects of genotype and diet (16). Another study reported the beneficial effects of increasing oleic acid intake on HDL-C, especially in allele R carriers (46). Also, in the current study, higher vitamin C intake in R allele carriers significantly reduced the risk of high cholesterol and increased the odds of low HDL-C, which may have occurred by chance because there was no interaction between vitamin C intake and rs662 polymorphism on TC and HDL-C. This result proposes the necessity of conducting prospective cohort studies based on interaction and with a larger sample size.

Although the exact mechanism for these interaction effects has not yet been discovered, these effects seem to be influenced by PON1 genotype-dependent enzyme activity (47). Changing the activity of this enzyme leads to altering the oxidation state in the body. This enzyme has an antioxidant and antiatherosclerotic function by hydrolyzing lipid peroxides and destroying pro-inflammatory molecules caused by LDL-C oxidation (48). Various studies have shown that enzyme activity is affected by both genetic and environmental factors (40, 49). Therefore, although a higher intake of antioxidant vitamins E and C can help to strengthen enzyme function, this effect is modulated by the genetic variants of rs662 polymorphism (40, 47).

The present study has strengths and limitations, which are briefly addressed. In terms of strengths, the current study is the first attempt to survey the interaction between PON1 rs662 polymorphism and antioxidant vitamins E and C on CAD severity and lipid profile that helps to prescribe personalized nutritional recommendations for the improvement and management of CVD risk in the future. In terms of limitations, due to the cross-sectional nature of the study design, it was not possible to conclude a causal association. This study was done only on patients in Iran, so it cannot be generalized to all races and ethnicities. Thirdly, due to budget limitations, it was not possible to measure the serum PON1 activity and investigate several polymorphisms at the same time, so it is difficult to talk about possible mechanisms. Fourth, FFQ is a memory-based dietary assessment method, so the results may not be accurate. Finally, two patients' genotype was not amplified.

## Conclusions

The novel finding of the present study was the existence of a significant interaction between rs662 polymorphism and vitamin C intake. More specifically, R allele carriers with lower vitamin C intake were susceptible to higher LDL-C. The findings of such studies could be critical for clinical diagnosis, gene-based therapy, and providing individualized nutritional recommendations. Although, mechanism-based studies with larger sample sizes are needed in this field.

## Data availability statement

The original contributions presented in the study are included in the article/Supplementary material, further inquiries can be directed to the corresponding author.

## Ethics statement

The present cross-sectional study was approved by the Ethics Committee of Isfahan University of Medical Sciences (Ethical Approval Code: IR.MUI.RESEARCH.REC.1400.200). The patients/participants provided their written informed consent to participate in this study.

## Author contributions

GHA and AS-A contributed to the conception and design of the study. AF and MD performed the statistical analysis. MD wrote the first draft of the manuscript. MVM and SMS contributed to the data collection. All authors contributed to manuscript revision, read, and approved the submitted version.

## Supplementary material

Supplementary Figure S1 Flow diagram of study participants.
