# Supplementary material for: Joint effects of paraoxonase 1 rs662 polymorphism and vitamins C/E intake on coronary artery disease severity (Gensini and SYNTAX scores) and lipid profile in patients undergoing coronary angiography
Source: Front Nutr. 2023 Feb 2;9:1097411. doi: 10.3389/fnut.2022.1097411 (PMC9932536; doi:10.3389/fnut.2022.1097411)
Supplement: Supplementary Figure S1 — Flow diagram of study participants. [file Presentation_1.PPTX]

## Slide 1
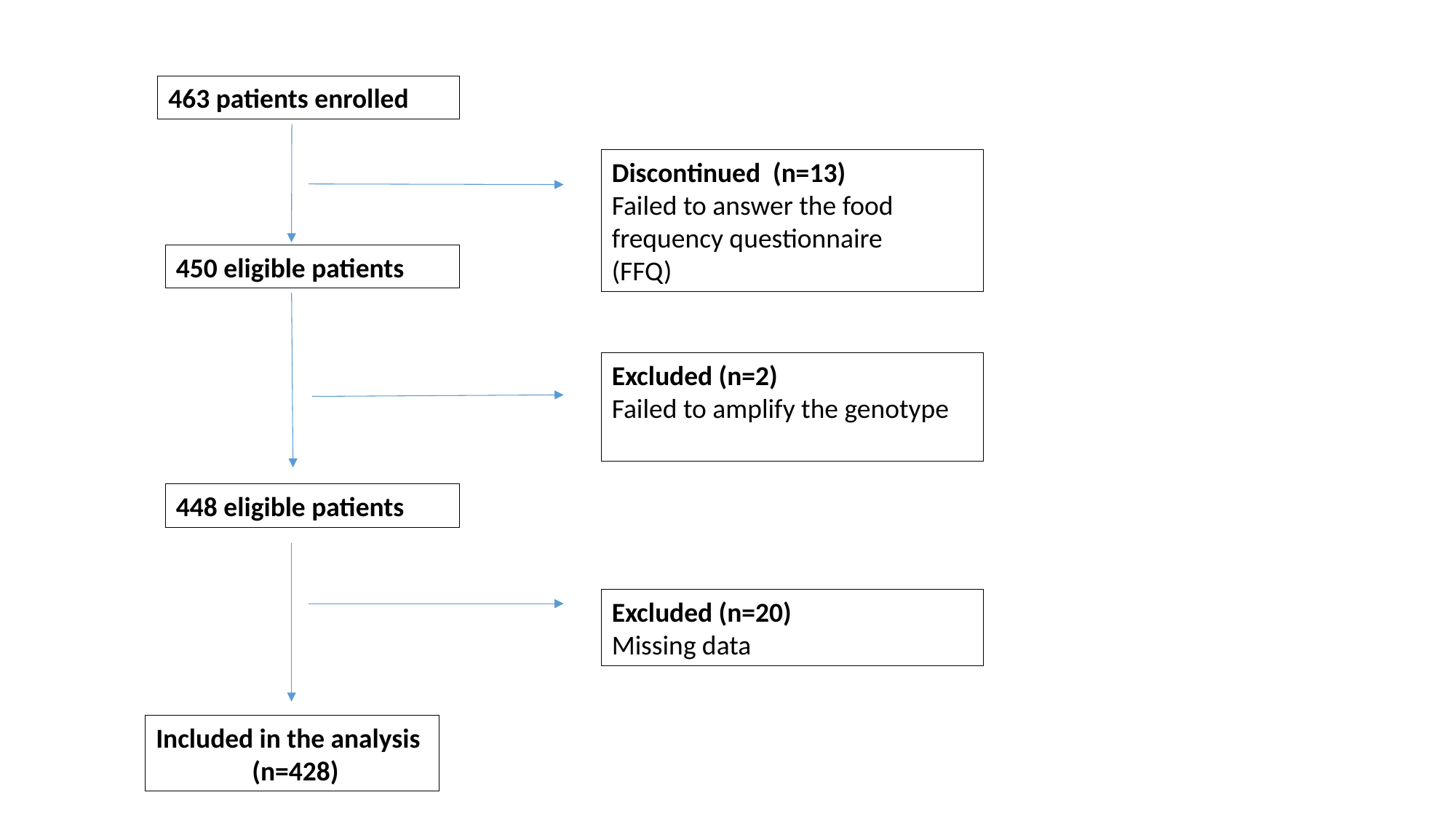

463 patients enrolled
Discontinued (n=13)
Failed to answer the food frequency questionnaire
(FFQ)
450 eligible patients
Excluded (n=2)
Failed to amplify the genotype
448 eligible patients
Excluded (n=20)
Missing data
Included in the analysis
 (n=428)
